# The longitudinal changes of BOLD response and cerebral hemodynamics from acute to subacute stroke. A fMRI and TCD study

**DOI:** 10.1186/1471-2202-10-151

**Published:** 2009-12-20

**Authors:** Claudia Altamura, Matthias Reinhard, Magnus-Sebastian Vry, Christoph P Kaller, Farsin Hamzei, Fabrizio Vernieri, Paolo Maria Rossini, Andreas Hetzel, Cornelius Weiller, Dorothee Saur

**Affiliations:** 1 Neurologia Clinica, Università Campus Bio-Medico di Roma, Italy; 2 Associazione Fatebenefratelli per la Ricerca (AFaR): Dipartimento di Neuroscienze, Fatebenefratelli, Isola Tiberina, Rome, Italy; 3 Department of Neurology, University Medical Center Freiburg, Germany; 4 Casa di Cura San Raffaele - Cassino, Cassino, Italy

## Abstract

**Background:**

By mapping the dynamics of brain reorganization, functional magnetic resonance imaging MRI (fMRI) has allowed for significant progress in understanding cerebral plasticity phenomena after a stroke. However, cerebro-vascular diseases can affect blood oxygen level dependent (BOLD) signal. Cerebral autoregulation is a primary function of cerebral hemodynamics, which allows to maintain a relatively constant blood flow despite changes in arterial blood pressure and perfusion pressure. Cerebral autoregulation is reported to become less effective in the early phases post-stroke.

This study investigated whether any impairment of cerebral hemodynamics that occurs during the acute and the subacute phases of ischemic stroke is related to changes in BOLD response.

We enrolled six aphasic patients affected by acute stroke. All patients underwent a Transcranial Doppler to assess cerebral autoregulation (Mx index) and fMRI to evaluate the amplitude and the peak latency (time to peak-TTP) of BOLD response in the acute (i.e., within four days of stroke occurrence) and the subacute (i.e., between five and twelve days after stroke onset) stroke phases.

**Results:**

As patients advanced from the acute to subacute stroke phase, the affected hemisphere presented a BOLD TTP increase (p = 0.04) and a deterioration of cerebral autoregulation (Mx index increase, p = 0.046). A similar but not significant trend was observed also in the unaffected hemisphere. When the two hemispheres were grouped together, BOLD TTP delay was significantly related to worsening cerebral autoregulation (Mx index increase) (Spearman's rho = 0.734; p = 0.01).

**Conclusions:**

The hemodynamic response function subtending BOLD signal may present a delay in peak latency that arises as patients advance from the acute to the subacute stroke phase. This delay is related to the deterioration of cerebral hemodynamics. These findings suggest that remodeling the fMRI hemodynamic response function in the different phases of stroke may optimize the detection of BOLD signal changes.

## Background

Neural tissue that survives the damaging effects of a stroke can reorganize in order to recover lost functions. The disinhibition of cortical activities can lead to the over-activation of areas belonging to the physiological neural network and of areas that are recruited to replace the functions of the damaged tissue. Alternatively, cerebral activation can be inhibited in brain regions that are remote from the damaged area (i.e., diaschisis) [1-3]. The impact of brain area over-recruitment on functional recovery was investigated by longitudinal studies that tracked the evolution of such plastic changes over time [4-6]. These studies found that there was an over-activation of certain brain areas (including supplementary areas and contra-lesional homologue areas) in the sub-acute phase followed by the progressive focusing of cerebral activation associated with clinical recovery [4-6]. In aphasic patients, this phenomenon was reported to have a tri-phasic trend from the acute to the chronic stage; the initially reduced cerebral activation was followed by an over-activation of the language cortical network in the subacute phase, which was followed by a progressive focusing of cerebral activation [7].

Although the use of fMRI allowed investigators to make significant progresses in understanding post-stroke cerebral plasticity, blood oxygen level dependent (BOLD) signals may be reduced [8-13], absent [14,15] or negative [16] in patients with cerebro-vascular disease. Cerebro-vascular diseases may also affect the shape of the hemodynamic response function (HRF) of BOLD signal. In particular, HRFs in such patients can have lower amplitudes, longer latency to peak intervals (time to peak = TTP), and deeper initial dips [9,16-19].

These findings were also reported in cases of preserved neuronal activity [14-16] and in relation to altered cerebral hemodynamics [9,11,14], which suggests that reduced BOLD signals might reflect the decrease of neuronal activation or be the consequence of neurovascular uncoupling. These results raise the question of whether the longitudinal changes of cerebral activation that are found during early post-stroke phases [4-7] are due in part to cerebral hemodynamic impairment.

Cerebral hemodynamics are characterized by the following two distinctive properties: autoregulation (i.e., the maintenance of relatively constant blood flow despite changes in arterial blood pressure (ABP) and perfusion pressure) and vasomotor reactivity (i.e., the potential for cerebral vessels to dilate subsequent to hypercapnia). The assessment of cerebral vasomotor reactivity requires the patient's cooperation since hypercapnia is induced by holding one's breath or by inhaling CO_2 _enriched air. In addition, this test is potentially harmful for acute stroke patients since hypercapnia may increase ABP. By contrast, autoregulation can be non-invasively evaluated by correlating the spontaneous oscillations of ABP and cerebral blood flow velocity (CBFV). This does not require any cooperation from the patient [20,21]. This correlation is measured by calculating a coefficient named Mx index [20,21]. A high Mx index indicates a dependence of CBFV on ABP that is attributable to autoregulatory impairment.

Cerebral autoregulation was reported to worsen between the acute and subacute stroke phases [20,21]. This phenomenon could be explained by arteriolar dysfunction that develops at the ischemic site and spreads to remote areas later in the post-stroke interval. It was hypothesized that a vicious circle can start in the peri-infarct area by spreading local acidosis, and then is amplified by reperfusion (either spontaneously or induced by thrombolysis) with a consequent dysautoregulation [21,22]. In addition, bursts of oxidative stress induced by cerebral ischemia lead to profound alterations in cerebro-vascular regulation. In particular, reactive oxygen species can impair endothelial NO-mediated responses, vasodilation (mediated by potassium channel activation), and vasoconstrictor mechanisms [23]. Finally, hemispheric strokes can damage the central autonomic network thereby altering the neural control mechanisms of cerebral vessels [24].

Despite early impairment of cerebral hemodynamics, longitudinal changes of BOLD signal HRF in the acute and subacute stroke phases have never been explored. One hypothesis is that the deterioration of cerebral hemodynamics in early phases following a stroke can affect BOLD signal HRF.

This study investigated whether the impairment of cerebral hemodynamics that occurs as patients advance from the acute to the subacute stroke phase is related to changes in the BOLD signal HRF.

To test this hypothesis, we evaluated cerebral autoregulation (Mx index) and the amplitude and peak latency (TTP) of BOLD responses to auditory stimuli in the auditory cortex of 6 aphasic patients during the acute and the subacute phases of stroke. At the time of recruitment, the patients were taking part in two ongoing longitudinal fMRI studies on language recovery.

## Results

### Clinical findings

**Figure 1:**
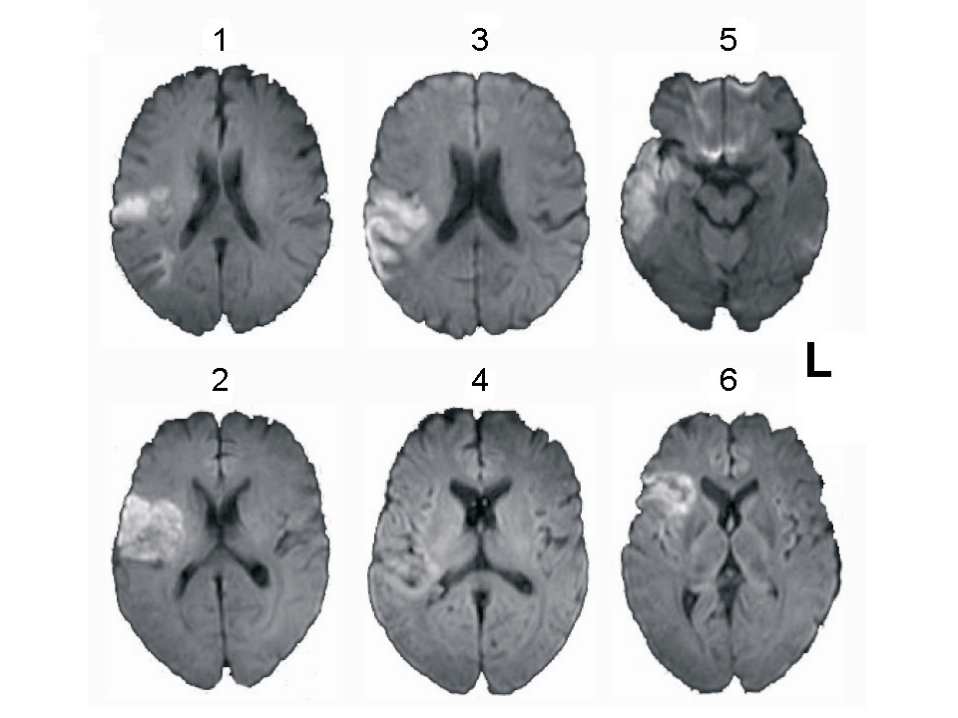
**Ischemic Lesions**. Axial Diffusion Weighted MR images of enrolled patients. The left side of the figure corresponds to the left side of the brain.

Table 1 summarizes our patients' clinical characteristics. Figure 1 shows diffusion-weighted MRIs at the maximum infarct volume level.

**Table 1:** Patients' clinical characteristics.

Pt	Age	AH	Clinical Symptoms	NIHSS Exp 0	Lesion site	Stenosis ICA (%)		Vessel Occlusion	Trombolysis	Risk factors
						AH	UH			
1, ♂	66	L	Aphasia	8	PC	0	0	MCA-branch	Y	Hypert, smoke

2, ♂	69	L	Aphasia/Hemiparesis FB	14	FC, PC	0	0	MCA-branch	Y	AF

3, ♂	63	L	Aphasia	6	FC, PC	0	0	MCA-branch	Y	Hypert, AF

4, ♂	46	L	Aphasia	6	PC	100	0	M2	N	Hypert, smoke

5, ♀	72	L	Aphasia	3	TC	0	0	MCA-branch	Y	Hypert, Chol

6, ♀	44	L	Aphasia	7	FC	0	0	MCA-branch	Y	Hypert

*Pt:*patient; ♂: man, ♀: woman; *AH*: affected hemisphere, UH: unaffected hemisphere; *L *= left, *R *= right; *Clinical Symptoms: *FBC = facial/brachial/crural; *NIHSS Exp 0*: NIH stroke scale at emergency room admission; *Lesion site: *BG = basal ganglia, IC = internal capsula, PC = parietal cortex, FC = frontal cortex, TC = Temporal Cortex; *Stenosis ICA *= internal carotid artery; *Vessel Occlusion: *MCA = Middle Cerebral Artery, M2 = M2 segment of middle cerebral artery, *Trombolysis*: Y = yes, N = no; *Risk factors*: hypert = Hypertension, AF = atrial fibrillation, Smoke = cigarette smoking, Chol = hypercholesterolemia.

Clinical status significantly improved between the first (Ex 1) the second (Ex 2) examination (Wilcoxon test, p = 0.04). None of the parameters examined in the fMRI or TCD studies was related to vascular risk factors or clinical outcome (Spearman's rho, consistently p > 0.2).

### Ultrasound findings

Cerebral hemodynamic parameters are listed in Table 2. CBVF did not differ significantly between the two hemispheres in either the acute (Wilcoxon test, p = 0.3) or in subacute stroke phase (p = 0.2). No variation of CBVF was observed between Ex 1 and Ex 2 in the two hemispheres (affected hemisphere (AH), p = 0.17; unaffected hemisphere (UH), p = 0.11).

**Table 2:** fMRI and Cerebral hemodynamics parameters from the acute to the subacute phase of stroke

	Pt	Ex 1	Ex 2	NIHSS		AH								UH							
						
						TTP		Ampl		CBFV		Mx index		TTP		Ampl		CBFV		Mx index	
				
				Ex 1	Ex 2	Ex 1	Ex 2	Ex 1	Ex 2	Ex 1	Ex 2	Ex 1	Ex 2	Ex 1	Ex 2	Ex 1	Ex 2	Ex 1	Ex 2	Ex 1	Ex 2
**Parad 1**	1	1	5	6	3	5.48	6.57	0.86	0.56	48.6	56.9	0.01	0.54	5.48	6.57	0.89	0.50	50.1	45.9	-0.15	0.52
	
	2	2	8	14	13	5.48	6.57	0.56	0.42	39.5	33.1	0.04	0.41	5.48	6.57	0.58	0.48	43.2	27.1	-0.05	0.28
	
	3	2	5	4	2	5.48	7.67	0.62	0.52	41.8	30.6	-0.12	0.42	5.48	6.57	0.89	0.49	47.7	35.8	-0.10	0.36

**Parad 2**	4	3	10	5	4	-	-	-	-	50.3	53.8	0.14	0.27	5.52	5.52	0.25	0.33	51.4	43.7	0.42	0.09
	
	5	4	12	3	1	5.52	6.44	0.45	0.17	57.8	46.2	0.26	0.25	5.52	5.52	0.19	0.32	36.1	45.6	0.12	0.23
	
	6	1	9	3	3	5.52	6.44	0.53	0.34	62.7	53.1	0.14	0.66	5.52	6.44	0.44	0.53	77.9	55.4	0.21	0.57

Mean (SD)		2.2 (1.2)	8.2 (2.8)	5.8 (4.2)	4.3 (4.4)	5.50 (0.02)	6.74 (0.53)	0.60 (0.16)	0.40 (0.16)	50.12 (8.96)	45.62 (11.25)	0.08 (0.13)	0.43 (0.16)	5.50 (0.02)	6.20 (0.53)	0.54 (0.30)	0.44 (0.09)	51.07 (14.27)	42.25 (9.70)	0.08 (0.22)	0.34 (0.18)
		
				**p = 0.04**		**p = 0.04**		p = 0.06		p = 0.17		**p = 0.046**		p = 0.059		p = 0.46		p = 0.11		p = 0.09	
				

*Parad 1 and 2: *Language paradigm 1 and 2 respectively; *Ex 1 and Ex 2*: days from stroke onset; *NIHSS *NIH stroke scale at the first examination time (Ex 1) and at the second examination time (Ex 2); *TTP: *time to peak expressed in seconds and *Ampl: *Peak Amplitude of BOLD.; *AH*: affected hemisphere, UH: unaffected hemisphere; *CBFV*: mean MCA cerebral blood flow velocity expressed in cm/s; *Mx index: *correlation coefficient between CBFV and arterial blood pressure; *Mean (SD): *mean values (Standard deviation); in the bottom raw p values for comparison of each measure from Ex 1 to Ex 2 are reported, and evidenced in bold in case of statistical significance.

At Ex 1, the Mx index did not differ between the hemispheres (Wilcoxon test, p = 0.8), though the Mx index was higher at Ex 2 (i.e., worse cerebral autoregulation) in the AH as compared with the UH (p = 0.027). The Mx index increased between examinations in the AH (Wilcoxon test, p = 0.046) and not significantly in UH (p = 0.09)

### fMRI findings

Table 3 shows TTP and amplitude values that were measured in both hemispheres of the control subjects. Table 2 shows TTP and amplitude values that were measured in the AH and UH of stroke patients at Ex 1 and Ex 2. Because of an internal carotid occlusion, the fMRI failed to detect auditory cortical activation in the AH of patient 4.

**Table 3:** Parameters of Hemodynamic Response Function of BOLD signal in controls

			TTP		Ampl					TTP		Ampl	
					
	Sb	Age	R	L	R	L		Sb	Age	R	L	R	L
**Paradigm 1**	1	60	5.48	5.48	0.72	0.52	**Paradigm 2**	7	66	5.52	5.52	0.31	0.28
			
	2	69	5.48	5.48	0.62	0.58		8	45	5.52	5.52	0.31	0.42
			
	3	71	5.48	5.48	0.57	0.85		9	59	5.52	5.52	0.49	0.41
			
	4	56	5.48	5.48	0.59	0.68		10	41	5.52	5.52	0.44	0.49
			
	5	58	5.48	5.48	0.88	0.93		11	57	6.44	6.44	0.50	0.67
			
	6	46	5.48	5.48	0.24	0.22		12	52	5.52	5.52	0.31	0.37

Mean (SD)		60 (9)	5.48	5.48	0.60 (0.19)	0.63 (0.23)			53 (9)	5.67	5.67	0.39 (0.09)	0.44 (0.13)
					

**Figure 2:**
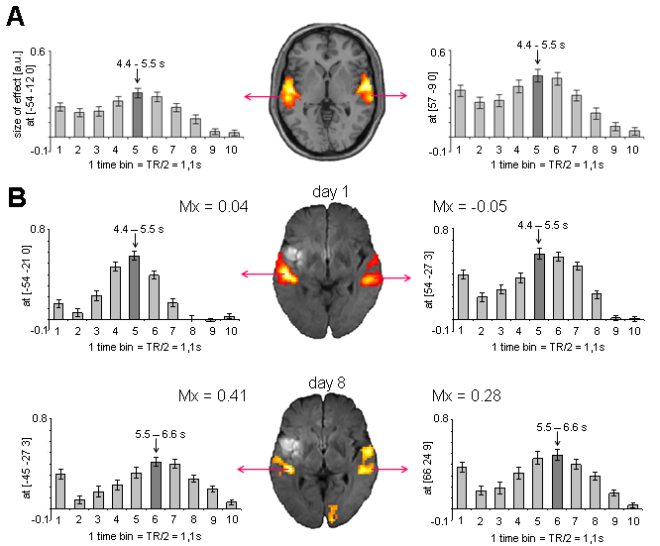
**BOLD signal in controls and patient 2 performing language paradigm 1**. **(A) **HRF extracted from the peak voxel in the bilateral auditory cortex of six healthy control subjects performing language paradigm 1. Plots represent the mean contrast estimate across subjects (y-axis) within each time bin (x-axis). The dark grey bar indicates the time bin with the highest contrast estimate as an estimation of the TTP latency. **(B) **HRF extracted from the peak voxel in the bilateral auditory cortex of patient 2 at day 2 (upper row) and day 8 (lower row). Plots represent mean contrast estimate across stimuli (x-axis) within each time bin (y-axis). From day 1 to day 8, TTP latency increased (from time bin 5 to time bin 6) and amplitude decreased slightly in both hemispheres.

**Figure 3:**
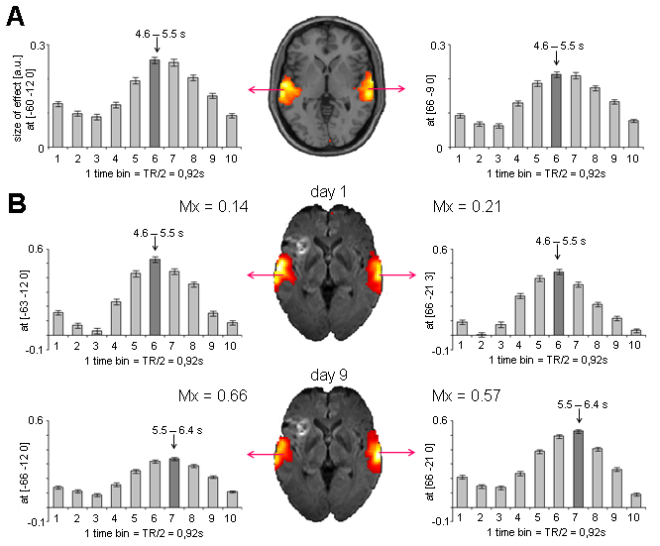
**BOLD signal in controls and patient 6 performing language paradigm 2**. **(A) **HRF extracted from the peak voxel in the bilateral auditory cortex of six healthy control subjects performing language paradigm 2. Plots represent the mean contrast estimate across subjects (y-axis) within each time bin (y-axis). The dark grey bar indicates the time bin with the highest contrast estimate as an estimation of the TTP latency. **(B) **HRF extracted from the peak voxel in the bilateral auditory cortex of patient 6 at day 1 (upper row) and day 9 (lower row). Plots represent mean contrast estimate across stimuli (x-axis) within each time bin (y-axis). From day 1 to day 9, TTP latency increased in both hemispheres (from time bin 6 to time bin 7) and amplitude decreased in the lesioned hemisphere.

*Paradigm 1 and 2: *Language paradigm 1 and 2 respectively; *TTP: *time to peak expressed in seconds and *Ampl: *Peak Amplitude of BOLD; *R*: right hemisphere; L: left hemisphere. Please note that differences in the mean amplitude values between table 3 and figures 2 and 3 are due to the fact, that in the table values were extracted from individual peak voxels, while in the figures they were extracted from the group peak voxel.

TTP measurements did not reveal differences between the two hemispheres at Ex 1 (Wilcoxon test, p = 0.9) or Ex 2 (p = 0.18). At Ex 2 TTP was delayed (p = 0.04) in the AH and in the UH with a trend toward significance (p = 0.059).

At both examination times, amplitude measurements did not reveal differences between hemispheres (Wilcoxon test, p > 0.2). Between Ex 1 and Ex 2, amplitude did not change in the UH (p > 0.4), while it tended to decrease with a trend toward significance in the AH (p = 0.06).

Figures 2 and 3 contain estimations of the hemodynamic response function (HRF) in six healthy subjects (panel A) and one representative patient (panel B) for language paradigms 1 and 2, respectively.

To analyze a possible correlation between TTP elongation and Mx increase, we first calculated the variation between Ex 1 and Ex 2 for each parameter. We found that BOLD peak delays correlated with worsening cerebral auto-regulation (Spearman's rho = 0.734; p = 0.01). This result was obtained after taking into consideration, through the use of a Generalized Estimating Equations model, both hemispheres and the within-subject dependence.

## Discussion

Our main finding is that the progressive worsening of cerebral hemodynamics is related to an increased latency of the BOLD signal HRF from the acute to the subacute stroke phase.

Increases in BOLD peak latency were previously observed in patients with cerebro-vascular stenotic disease [18,25] and in patients with chronic aphasia [19]. BOLD peak latency increases were also analyzed in relation to clinical outcome [17]. Different hypotheses can explain the findings in these studies. During activation tasks, delayed BOLD peak latency can be the effect of initial oxidative metabolism wherein an increase in deoxy-hemoglobin is followed by a slower increase in oxy-hemoglobin and regional cerebral blood flow. In patients suffering from internal carotid or intracranial steno-occlusive disease, this phenomenon could be due to the vasodilatation of the arterioles down-stream from the stenosis, which increases the regional cortical blood volume at rest. In this condition, an additional increase of blood flow during neural activation is necessary for the detection of a BOLD response. When evaluating fMRI activation in a damaged cortex, the TTP increase might depend on the increased oxygen extraction fraction due to decreased oxygen delivery during activation [26,27].

In contrast to previous studies reporting a TTP increase in the damaged cortex of chronic patients [17,19] or in asymptomatic patients with vascular steno-occlusive disease [18,25], we found increases in BOLD peak latency in preserved cerebral cortices (i.e., perilesional or those remote from the infarction) and in absence of steno-occlusive diseases. In our patients, a longer peak latency of BOLD HRF could be the consequence of a general deterioration of arteriolar endothelial/smooth muscle cell function, which impairs cerebral auto-regulation [21-23] and limits the increase of regional blood flow (and oxy-hemoglobin) produced by functional activation [26].

On the other hand, neither BOLD HRF nor cerebral auto-regulation was altered in the acute stroke phase. This suggests that an alteration of cerebral hemodynamics alone cannot explain the impairment of BOLD signal in acute stroke patients. Decreased fMRI activation, which was previously reported in the acute stroke phase [2,6,7], could actually reflect lower levels of neuronal firing also in cortical areas distant from the ischemic lesion. The following potential reasons for such reduced neuronal function can be hypothesized: an initial "stunned" brain phenomenon that is functioning as a protective mechanism [28]; cellular and metabolic changes; prolonged hypoperfusion despite recanalization; and incomplete infarction with neuronal dysfunction after critical hypoperfusion [29,30]. An alternative explanation may involve the functional-anatomic location of the lesion and its participation in a neuronal network (i.e., "diaschisis") [31].

Previous studies have reported that hemodynamic impairment can affect fMRI signal detection [9,11,14]. In the chronic stroke phase, BOLD HRFs can have decreased amplitudes and longer latencies to peak [17,19]. The present study confirms these findings and reports for the first time the dynamic variations of BOLD signals from the acute to the subacute stroke phase as they relate to cerebral hemodynamic deterioration. This may provide additional knowledge for the interpretation of early changes in post-stroke fMRI activation.

New fMRI studies are targeting early cerebral reorganization within the acute and sub acute stroke phases, probably responsible for initial clinical recovery and for long-term outcome. However, our findings recommend caution when interpreting results from longitudinal studies. Changes in BOLD activation could be either the result of cerebral reorganization or the effects of hemodynamic impairment over time. In particular, if the HRF of BOLD signal detected in stroke patients has a TTP different from that normally assumed, the analysis fMRI data might be invalidated. The main limitation of our study is the small number of patients enrolled. In fact, this may have lead to an underestimation of other longitudinal changes of BOLD HRF (i.e., amplitude). In addition, since hypertension and other vascular factors can influence cerebral hemodynamics and thereby affect BOLD signals [32], a control population with vascular factors comparable to our patients would allow us to better compare TTP values between the two groups. We cannot exclude the possibility that the observed increases in TTP in our patients were the consequence of their additional vascular ailments rather than the result of the stroke itself. Similarly, in order to assess whether TTP could be considered a stable measure in different examinations, control subjects should have undergone fMRI at two different times.

## Conclusions

Our data demonstrate a longitudinal change in BOLD HRF that is possibly due to the sub-acute impairment of cerebral hemodynamics. Our findings suggest that remodeling the fMRI HRF and adapting other parameters (e.g., stimulus duration or signal acquisition time) for different stroke phases may optimize the detection of BOLD signal changes. In addition, an integrated analysis that includes neurophysiological techniques or hemodynamic evaluation should be used also in longitudinal studies that investigate cerebral plastic reorganization via metabolic signal based imaging methods.

Further studies are needed to confirm our findings for different stroke types and vascular territories and to assess the extent the mean TTP delay in the sub-acute stroke phase in a larger population. This would be useful for adapting the hemodynamic function that underlies the BOLD-signal in the analysis of cerebral activation data from patients in the subacute post-stroke phase.

## Methods

### Patients

Six patients (4 men; mean age 60.0 ± 12.0 years) with acute ischemic stroke within the middle cerebral artery (MCA) territory presenting with aphasia were enrolled in this study. The diagnosis of ischemic stroke was based on clinical criteria and on the results of diffusion weighted MRI at admission (figure 1). The site of vessel occlusion was demonstrated using Time-of-Flight MR angiography. Vascular risk factors and carotid ultrasound findings were assessed for each patient by reviewing their clinical charts. Tables 1 and 3 detail the patients 'clinical data and the examination times. Since leukoencephalopathy may affect BOLD signals [32], we excluded patients whose MRI rated >4 for the "Age Related White Matter Changes" score [33]. Patients with a poor insonation of MCAs at the temporal bone windows were excluded since TCD examinations could not be performed.

### Controls

The hemodynamic response function (HRF) was estimated in 12 healthy, age-matched control subjects (8 men, mean age of 57 ± 9.4 years). Reference values for hemodynamic parameters were derived from a previously analyzed older adult control population (mean age 63 ± 9 years) [34].

The local ethics committee approved the study (267/05), and informed consent was obtained from all subjects.

### Experimental design

Experimental examinations were carried out at the acute phase (Ex 1; i.e., within four days of stroke occurrence) and the subacute phase (Ex 2; i.e., between five and twelve days after stroke occurrence and at least two days after Ex 1). The examinations included:

1. Transcranial Doppler (TCD) to evaluate (i) patency and blood flow velocity of middle cerebral arteries (MCAs) and (ii) cerebral autoregulation.

2. fMRI study to assess BOLD signal HRF in bilateral auditory cortices in response to two language paradigms.

The autoregulation and fMRI sessions were performed within six hours of each other.

Neurological status was assessed with the NIH stroke scale (NIHSS) at admission (Ex 0) and before each measurement (Ex 1 and Ex 2).

#### Cerebral hemodynamics evaluation

Measurements were performed with subjects in a supine position with slight to moderate elevation of the upper body. CBFV in both MCAs was measured by TCD with 2 MHz transducers attached to a head frame (TC2-64, EME, Germany). Continuous ABP recording was achieved via a servocontrolled finger plethysmograph (Finapres, USA). End-tidal CO_2 _partial pressure was measured in mm Hg with an infrared capnometer (Normocap Datex, Finland) during nasal expiration. After establishing stable values, a data segment of ten minutes was recorded as patients breathed spontaneously.

#### Cerebral autoregulation

In order to grade cerebral autoregulation, we used the previously described correlation coefficient method, which makes use of spontaneously occurring fluctuations in ABP and CBFV [20]. This approach is well established in neurocritical care and has been validated against static autoregulation measurements [35]. It is based on the simple assumption that decreasing cerebral autoregulation leads to an increasing correlation between fluctuations in CBFV and ABP (i.e., CBFV depends increasingly on fluctuations in ABP). To quantify this correlation, mean values of ABP and CBFV raw data were first averaged over three seconds. For 20 of these three-second averages (i.e., for one-minute periods), Pearson's correlation coefficients between the mean ABP and CBFV were calculated. The resulting sets of one-minute correlation coefficients gained from the entire time series were then averaged, yielding the autoregulatory index Mx. Mx increases with decreasing dynamic autoregulatory capacity. According to reference ranges defined in an elder population [34], Mx ≥ 0.46 corresponds to exhausted cerebral autoregulation.

#### fMRI paradigms

In both paradigms, stimuli were spoken by a female voice and recorded by Cool Edit software. Stimuli were presented binaurally through MR compatible headphones. Patients were asked to listen carefully and press a button at the end of each stimulus to ensure alert listening. In the present study, we were only interested in auditory cortex activation in response to speech stimuli as compared with background noise. Since our Department of Neurology enrolled stroke patients in different fMRI studies, the six patients included in this study underwent two different fMRI language paradigms. However, both paradigms were event-related experiments that differed only in the number of stimuli and sessions.

##### Language paradigm 1

In an auditory comprehension task, we presented 30 normal speech stimuli (German sentences, e.g., "Der Pilot fliegt das Flugzeug" [English translation: "The pilot flies the plane"]), 30 stimuli of pseudo speech, which was derived from normal speech stimuli by exchanging phonemes (e.g., "Ren simot plieft mas kugireug", [English translation is not possible]), and 30 stimuli of reversed speech (e.g., "guezgulf sad tgeilf tolip red", [English translation is not possible]). The reversed speech stimuli were using the Cool Edit software. Stimuli duration ranged between 1730 and 2720 ms. Stimuli were presented binaurally in a pseudo randomized order with an inter-stimulus interval that varied between 3000 and 6000 ms. Stimuli were assigned to a single nine minute long session.

##### Language paradigm 2

In a modified version of paradigm 1, we presented 92 stimuli of normal speech (German sentences, e.g., "Der Pilot fliegt das Flugzeug" [English translation. "The pilot flies the plane"]) and of reversed speech (e.g., "guezgulf sad tgeilf tolip red", [English translation is not possible]). Stimuli were presented binaurally in a pseudo randomized order with an inter-stimulus interval that varied between of 3000 and 6000 ms. Stimuli were assigned to six sessions, resulting in a total scanning time of 21 minutes (for details see ref 7).

#### MRI data acquisition

Functional and structural MRI data from all subjects were acquired on a 3 T Siemens TIM Trio scanner with a standard head coil.

##### Diffusion weighted imaging (DWI)

Scans were obtained using a standard in-house stroke DWI sequence (23 slices, matrix 128 × 128 pixel^2^, voxel size 1.8 × 1.8 × 6 mm^3^, TR = 3.1 s, TE = 79 ms, flip angle = 90°).

##### Functional MRI

In cases where the sequence specifications differ between paradigms, values for language task 1 and 2 are given in parentheses. A total of 1 × 260 (6 × 115) scans per examination with 36 (32) axial slices covering the whole brain was acquired in interleaved (descending) order using a gradient echo echo-planar (EPI) T2*-sensitive sequence [resolution = 3 × 3 × 3 mm^3^, TR = 2.19 (1.83) s, TE = 30 (25) ms, flip angle = 75° (70°), matrix = 64 × 64 pixel^2^]. During reconstruction, scans were corrected for motion and distortion artifacts based on a reference measurement.

##### MP-RAGE

A high-resolution T1 anatomical scan was obtained (160 slices, voxel size = 1 × 1 × 1 mm^3^, TR = 2.2 s, TE = 2.6 ms, FOV = 160 × 240 × 240 mm^3^) for spatial processing of the fMRI data.

#### fMRI Data analysis

fMRI data were analyzed with SPM5 (http://www.fil.ion.ucl.ac.uk/spm).

##### Preprocessing

Data were pre-processed using standard routines implemented in SPM5. In both experiments, slices were first corrected for different signals acquisition times by shifting the signal measured in each slice relative to the acquisition of the middle slice. Volumes were then spatially normalized to the Montreal Neurological Institute (MNI) reference brain using non-linear normalization parameters that were estimated during segmentation of the coregistered T1 anatomical scan [36]. All normalized images were then smoothed using an isotropic 9-mm Gaussian kernel to account for inter-subject differences. Data were motion corrected during acquisition using the method introduced by Zaitsev et al. [37]

##### Finite impulse response (FIR) analysis

The time course of the hemodynamic BOLD response in both hemispheres was estimated using FIR analyses as implemented in SPM5. Onsets of auditory stimuli were convolved with a set of ten successive basis functions, which resulted in ten temporally aligned regressors for each condition. Each single basis function estimated the size of the BOLD signal for a specific time window of length TR/2. Altogether, the condition-specific sets of the ten basis functions covered a total post stimulus time of 10.95 s and 9.2 s for paradigms 1 and 2, respectively (see Figure 2 and 3). F-contrasts were computed across all ten basis functions. In the peak voxels within both hemispheres, parameter estimates were extracted for each basis function. In single subject analyses on study patients, parameter estimates represent the averaged effect size across stimuli, while in random effects group analyses on controls, parameter estimates represent the averaged contrast estimate across subjects. Since parameter estimates resembled normalized values, comparisons across subjects were valid. For comparison across paradigms, contrast estimates were divided by the number of sessions (i.e. in case of paradigm 2 by factor 6). The time bin with the highest contrast estimate was used as an approximation of the TTP, and the contrast estimate itself reflects the amplitude of the HRF.

#### Statistical analysis

Statistical analysis was carried out using SPSS 17.0 software. Nonparametric tests (e.g., Wilcoxon signed-rank test for paired data, Spearman test) were used to compare a patient's data from the two hemispheres, to evaluate their variations over time, and to assess possible correlations. Since the sample size was small, we grouped data from both hemispheres in order to assess the correlation between TTP and Mx index. This was done using Generalized Estimating Equations (GEE) models, which allowed us to account for repeated measurements within subjects (in this case, the measurements from two hemispheres). We reported nominal p values and considered p values of p < 0.05 to be statistically significant. We did not adjust p values for multiple comparison adjustments since they would have significantly reduced the ability to detect interesting correlations within this small sample.

## List of abbreviations

**BOLD: **blood oxygenated level dependent, **fMRI: **functional magnetic resonance imaging, **TCD: **Transcranial Doppler, **TTP: **time to peak, **Ex 1: **examination in the acute phase, **Ex 2: **examination at the subacute phase, **NIHSSri: **National Institute of Health Stroke Scale recovery index, **AH: **affected hemisphere, **UH: **unaffected hemisphere, **HRF: **hemodynamic response function, **MCA: **middle cerebral artery, **CBFV: **Cerebral blood flow velocity, **ABP: **arterial blood pressure, **FIR: **finite impulse response.

## Authors' contributions

CA designed the research, carried out ultrasound examinations, and wrote the manuscript. MR designed the research, analyzed the data, and was involved with interpreting the data and revising the manuscript. MSV carried out motor fMRI studies and analyzed fMRI data. CPK analyzed HRF fMRI data, FH designed the research, CW, AH, FV and PMR interpreted the data and made critical revision of the manuscript, and finally, DS carried out language fMRI, analyzed fMRI data, and wrote the manuscript. All the authors erad and approved the final manuscript.
